# Early growth response protein 1 and dual specificity protein phosphatase 1 are involved in down-regulation of allergic responses

**DOI:** 10.1186/2045-7022-3-S2-P26

**Published:** 2013-07-16

**Authors:** Kornel Golebski, Danielle van Egmond, Esther de Groot, Wytske Fokkens, Cornelis van Drunen

**Affiliations:** 1 Academic Medical Center, ENT, Amsterdam, the Netherlands

## Background

The airway epithelium is accepted as an active player in immune responses. Besides its role as physical barrier towards invading pathogens and irritants, epithelium also affects the outcome of the immune response by the production of various pro-inflammatory mediators. We have previously shown that nasal epithelial cells are able to respond to exposure to house dust mite (HDM) allergen and that this response is different for epithelial cells isolated from healthy or from allergic individuals. Expression profiling in allergic individuals relative to healthy ones reveals genes that are permanently activated (e.g. NFKB-1, FOSL-1 and JUN) and genes that fail to be up-regulated (e.g. DUSP-1, EGR-1). As EGR-1 and DUSP-1 have been implicated in the down-regulation of inflammatory responses, we hypothesize that failure of up-regulation of DUSP-1/EGR-1 after exposure to HDM in allergic individuals could be responsible for the sustained activation of the allergic response.

## Methods

We characterized regulatory responses triggered by allergen and viral stimulation in airway epithelium and the contribution of EGR-1 or DUSP-1 to these responses. The parent human bronchiolar cell line (NCI-H292) together with two mutant cell lines with silenced EGR-1 or DUSP-1 were exposed to HDM or poly(I:C) in a time course of 96 hours. Expression levels of selected transcription factors and cytokines were quantified by the real-time PCR and ELISA.

## Results

Knock down of EGR-1 significantly enhanced and sustained the production of cytokines (e.g. IL-6, IL-8) after both HDM and poly(I:C) stimulation. The DUSP-1 knock down resulted in enhanced and sustained cytokines production after HDM stimulation. Additionally, in the wild type cell line, we observed a two-phase temporal response after HDM exposure, with EGR-1, DUSP-1, ATF-3 induced rapidly and reaching maximal expression not later than 1 hour after stimulation, while other genes and cytokines reached their maximal expression 4 hours after induction. This early indication of EGR-1, DUSP-1 and ATF-3 is compatible with the notion of an allergen c.q. viral-induced negative feedback loop of the inflammatory response. Furthermore, the high degree of overlap between the poly(I:C) and the HDM response suggests a potential mechanism of viral induced allergic exacerbations.

## Conclusions

Failure of EGR-1 or DUSP-1 up-regulation in allergic individuals could be responsible for the prolonged activated state observed in vivo.

